# Round Spermatid Injection (ROSI) as a Last Resort in High-Risk Azoospermia: Chain of Outcome Metrics and Real-World Safety Signals

**DOI:** 10.3390/jcm15072771

**Published:** 2026-04-07

**Authors:** Ümran Karabulut Doğan, Erhan Hüseyin Cömert, Tuğçe Baykara, Mustafa Taş, Yusuf Gençten, Telal Doğruel, Ozan Doğan

**Affiliations:** 1 Department of Obstetrics and Gynecology, Acıbadem Kayseri Hospital, Melikgazi 38140, Kayseri, Turkey; 2 Private Clinic, Obstetrics and Gynecology, Istanbul 34367, Turkey; erhan.comert@hotmail.com; 3 IVF Center, Department of Obstetrics and Gynecology, Acıbadem Kayseri Hospital, Melikgazi 38140, Kayseri, Turkey; baykaratugce@gmail.com; 4 Health Sciences Faculty, Istanbul Nisantasi University, Istanbul 34398, Turkey; drmustafatas@yahoo.com; 5 Department of Urology, Acıbadem Kayseri Hospital, Melikgazi 38140, Kayseri, Turkey; yusufgencten@gmail.com; 6 Department of Obstetrics and Gynecology, Istanbul Medicalpark Hospital, Istanbul 34250, Turkey; telaldogruel@hotmail.com; 7 Department of Obstetrics and Gynecology, Istanbul Nisantasi Universitesi, Istanbul 34398, Turkey

**Keywords:** round spermatid injection, ROSI, azoospermia, oocyte activation, fertilization, blastocyst, chain-of-outcome, clinical pregnancy, feasibility, safety signal

## Abstract

**Background/Objectives:** Round spermatid injection (ROSI) is considered an experimental “last resort” option for couples with severe male factor infertility when mature spermatozoa cannot be obtained. We aimed to identify which stage of the clinical chain most strongly constrains overall success in routine practice and to describe the observed safety signal. **Methods:** We conducted a retrospective single-center cohort study of 221 consecutive ROSI-evaluated cycles (2021–2024). Outcomes were analyzed using a chain-of-outcome framework with explicit denominators: cycle-level feasibility (≥1 injected oocyte), two pronuclei (2PN) formation per injected oocyte, blastocyst development per 2PN, transfer per blastocyst cycle, and clinical pregnancy per transfer and per initiated cycle. Exact (Clopper–Pearson) 95% confidence intervals (CIs) were reported. **Results:** ROSI feasibility was observed in 5 of 221 initiated cycles (2.3%; exact 95% CI 0.7–5.2). Among the five transfer procedures performed after successful progression through upstream stages, clinical pregnancy occurred in four (80.0%; exact 95% CI 28.4–99.5). At the initiated-cycle level, overall clinical pregnancy was 4 of 221 cycles (1.8%; exact 95% CI 0.5–4.6). **Conclusions:** The overall effectiveness of ROSI remained low at the initiated-cycle level because very few cycles reached procedural feasibility and early attrition remained substantial. Conditional downstream outcomes may appear favorable only among the rare cycles reaching fertilization and transfer, while safety inference remains highly imprecise due to small denominators. Because only five cycles reached feasibility, all downstream conditional estimates remained highly unstable and sensitive to single-case variation.

## 1. Introduction

Round spermatid injection has occupied a narrow and controversial space in assisted reproduction for three decades. It is conceptually attractive for couples in whom mature spermatozoa cannot be obtained, yet it remains technically demanding and biologically constrained. Early commentary framed spermatid injection as an exceptional intervention rather than an extension of routine intracytoplasmic procedures. That framing emphasized two points that remain relevant today: first, the probability of success was expected to be low for most candidates; second, uncertainty was intrinsic and should be communicated as part of ethical clinical practice [1].

Clinical proof of concept soon established that fertilization, embryo development, and pregnancy could occur after the injection of immature male germ cells. The early report by Tesarik and colleagues demonstrated that the approach was not purely theoretical and could, in selected cases, reach a clinical endpoint [2]. Subsequent work described laboratory techniques, zygote development, and selected pregnancies after spermatid injection [3,4,5,6], while later synthesis continued to show marked heterogeneity and unstable outcomes across programs [7]. The literature therefore converged on a pragmatic conclusion: ROSI may enable pregnancy for a small subset, but the pathway is fragile and heavily dependent on circumstances that are not consistently reproducible.

Two intertwined explanations dominate contemporary interpretations. The first is a mechanism and centers on oocyte activation. Mature spermatozoa trigger physiological calcium oscillations that initiate a cascade required for pronuclear formation and early development. Round spermatids may fail to trigger this sequence adequately, making fertilization a dominant bottleneck even when injection is technically successful [8]. The second is technical and begins earlier than fertilization: the identification and selection of the intended cell type remain difficult and can introduce a failure mode that looks like biological incapacity but is, at least partly, procedural. Work focusing on spermatid identification has highlighted this vulnerability and its likely contribution to inconsistent fertilization [9]. These constraints explain why interventions that aim to improve outcomes frequently target activation and technique, including methods such as electrical stimulation and protocol refinements that reopened the discussion of ROSI in modern practice [10,11].

In parallel, the ethical and policy environment has remained cautious. Professional guidance has repeatedly described round spermatid injection (ROSI) and round spermatid nucleus injection (ROSNI) as experimental, emphasizing limited evidence and the need for explicit informed consent and careful reporting [12,13,14,15,16]. Contemporary ROSI-focused synthesis and newer comparative data have refined rather than resolved the picture: systematic reviews continue to show a marked heterogeneity across ROSI studies, narrative reviews highlight the persistent dependence on correct spermatid identification and laboratory expertise, and newer comparative data suggest that, even when fertilization or implantation may approach ICSI in selected non-obstructive azoospermia cases, overall live births remain lower [7,17,18]. Because activation-focused adjuncts remain central to current attempts at improvement, recent assisted oocyte activation syntheses are also relevant; however, the newer evidence suggests that any benefit is concentrated mainly in couples with low or failed fertilization, whereas evidence for embryo development, live birth, and safety remains limited, heterogeneous, or of low certainty [19,20,21,22]. This position has persisted partly because outcome heterogeneity makes it hard to generalize and partly because safety questions require very large denominators and long-term follow-up. Even when no adverse outcomes are observed in small series, the absence of events does not imply the absence of risk. Classical statistical reasoning shows that, with small samples, zero observed events remain compatible with meaningful underlying risk, and exact confidence-interval methods are preferred to convey uncertainty [23,24]. The broader assisted reproduction safety literature similarly emphasizes that birth defect and child health risk estimation is sensitive to confounding, underlying infertility, embryo-transfer strategy, and multiplicity, meaning that small studies cannot settle the question [25,26,27,28,29]. Epigenetic and imprinting concerns have also been discussed for assisted reproductive technologies and remain conceptually relevant when immature germ cells are used, adding another reason to why surveillance must extend beyond immediate pregnancy outcomes [30,31,32,33]. Contemporary cohorts reporting early childhood follow-up after ROSI provide useful context, yet available denominators remain limited for strong reassurance about uncommon or longer-term outcomes [34].

Despite decades of discussion, a persistent practical gap remains. Couples and clinicians do not only ask whether pregnancy is possible; they ask where the pathway fails and whether there is a rational basis to proceed. A single pregnancy rate cannot answer that question: it collapses a sequence of conditional events into one endpoint and hides the stage at which attrition concentrates. This limitation is highlighted in a systematic synthesis emphasizing heterogeneity and inconsistent reporting across ROSI studies [7]. A chain-of-outcome framework addresses the gap by quantifying stage-specific rates from feasibility through fertilization, embryo development, transfer, and clinical pregnancy.

In the present study, “high-risk azoospermia” refers to a clinical context in which mature spermatozoa suitable for conventional ICSI could not be identified or were considered unlikely to be retrievable within the relevant treatment pathway, leading ROSI to be considered as a last-resort option.

Therefore, this study aims to quantify stage-specific chain-of-outcome metrics for ROSI in a high-risk azoospermia cohort in routine practice and to report uncertainty for each stage using exact binomial confidence intervals. The objectives are to estimate cycle-level feasibility, fertilization expressed as two pronuclei (2PN) per injected oocyte, blastocyst development per 2PN, transfer procedure per blastocyst cycle, and clinical pregnancy per transfer procedure and per initiated cycle, and to describe the observed fetal anomaly signal with explicit zero-event uncertainty. We hypothesize that the overall effectiveness is primarily constrained by feasibility and early fertilization, whereas downstream outcomes reflect a highly selected subset that has already passed the dominant bottlenecks.

## 2. Materials and Methods

### 2.1. Study Design and Reporting Framework

**Figure 1:**
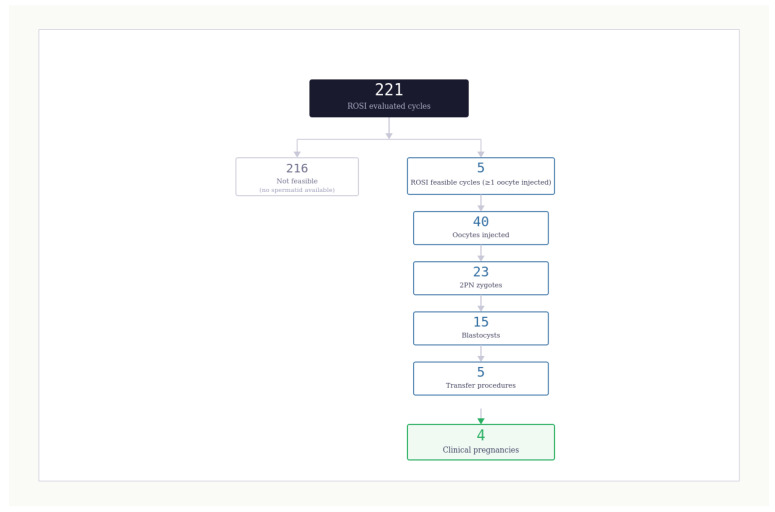
Flow diagram and chain counts for ROSI evaluated cycles. The flow diagram summarizes the 221 ROSI evaluated cycles and the progression through the chain of outcomes. ROSI feasibility is observed in five cycles, defined as at least one injected oocyte. Across feasible cycles, 40 oocytes are injected, yielding 23 two-pronuclei zygotes and 15 blastocysts. Five transfer procedures are performed, and four clinical pregnancies are recorded. Flowchart showing 221 evaluated cycles, 5 ROSI feasible cycles, 40 injected oocytes, 23 two-pronuclei zygotes, 15 blastocysts, 5 transfer procedures, and 4 clinical pregnancies.

A retrospective observational cohort is analyzed from a single tertiary assisted reproduction program. The cohort includes 221 consecutive cycles in which ROSI is evaluated within the clinical pathway during [2021–2024]. Reporting follows the Strengthening the Reporting of Observational Studies in Epidemiology guidance, with transparent denominators, explicit definitions, and a flow diagram summarizing selection and progression (Figure 1).

### 2.2. Participants and Clinical Context

Cycles are included when ROSI is considered as a last resort strategy in high-risk azoospermia after failure to identify mature spermatozoa suitable for conventional intracytoplasmic sperm injection. The dataset reflects the spectrum of clinical contexts in which ROSI enters discussion. The cohort was defined by cycles in which ROSI entered clinical evaluation or consideration within the institutional pathway, rather than only cycles in which ROSI was ultimately performed. Accordingly, obstructive azoospermia cases were retained if ROSI was considered after failure to identify mature spermatozoa suitable for conventional ICSI, even if no feasible ROSI was subsequently achieved. Azoospermia type is recorded as cryptozoospermia, non-obstructive azoospermia, or obstructive azoospermia. Sperm source is recorded as ejaculate, testicular sperm extraction (TESE), or microsurgical testicular sperm extraction (micro TESE). Genetic status is recorded as Y chromosome azoospermia factor microdeletions (AZF), 47,XXY, or not recorded. Female age and cycle descriptors such as number of mature oocytes are included as baseline descriptors. No idiopathic subgroup was available in the present dataset; all cycles were classified according to the recorded azoospermia category used in routine clinical documentation.

For the purposes of this study, high-risk azoospermia was defined pragmatically within the institutional pathway as azoospermic cases in which no mature spermatozoa suitable for conventional ICSI were identified despite routine clinical/laboratory evaluation, and ROSI therefore entered consideration as a rescue or last-resort strategy.

### 2.3. Embryologic Assessment and Spermatid Search Procedure

Round spermatid search was performed within the routine embryology laboratory workflow by experienced embryologists. As this study was retrospective, detailed laboratory process variables were not prospectively captured as standardized case-level data. Specifically, the number of high-power fields examined, duration of spermatid search, number of embryologists involved per case, and magnification threshold used for spermatid identification were not available in the database for any of the 221 cases (0/221 for all four parameters). Therefore, these variables could not be assessed quantitatively or compared across cases. This limitation has been acknowledged to avoid overinterpretation of laboratory feasibility assessment in the present cohort.

The retrospective database did not contain a structured variable specifying the immediate reason for infeasibility in the 216 non-feasible cycles (e.g., failure to identify sufficient round spermatids, procedural non-progression, or other laboratory/clinical barriers). Therefore, infeasibility could be quantified but not causally decomposed in the present study.

### 2.4. Outcome Definitions and Chain of Outcome Metrics

The chain of outcomes is defined as a sequence of clinically meaningful transitions with explicit denominators.

Feasibility was defined at the cycle level as the presence of at least one injected oocyte. This definition captures a practical reality that is often hidden in conventional reporting, namely that many cycles in which ROSI is evaluated do not reach an attempt to get an injection.

Fertilization is defined as two pronuclei (2PN) formation and is quantified per injected oocyte. The denominator is the number of injected oocytes across feasible cycles.

Embryo development is defined as blastocyst formation per two-pronuclei zygote. The denominator is the number of two-pronuclei zygotes.

Transfer is defined as the occurrence of a transfer procedure in a cycle with at least one blastocyst. The denominator is the number of feasible cycles that produced at least one blastocyst.

Clinical pregnancy is defined as an ultrasound-confirmed intrauterine gestational sac with or without fetal heartbeat, recorded in the clinical database after embryo transfer. If ultrasound confirmation is not available in a subset, the database definition used for clinical pregnancy is stated explicitly and applied consistently across all cycles.

The safety signal is captured as recorded fetal anomalies among clinical pregnancies. This is reported descriptively with explicit uncertainty.

### 2.5. Sample Size Rationale and Precision

The sample includes all consecutive ROSI-evaluated cycles in the study period, reflecting real-world practice rather than pre-planned enrollment. Precision was addressed explicitly. For feasibility, the denominator of 221 yields an exact confidence interval quantifying uncertainty in the underlying feasibility probability even with complete inclusion. For pregnancy per initiated cycle, the small number of events implies a wide uncertainty that was reported rather than masked. For safety, the pregnancy denominator is extremely small, and the absence of observed anomalies was interpreted through zero-event logic rather than reassurance [23,24].

### 2.6. Statistical Analysis

Stage-specific proportions were reported with exact binomial 95% confidence intervals using the Clopper–Pearson method [24]. Exact methods were used because several numerators and denominators were small. For outcomes with zero observed events, uncertainty was communicated explicitly, and zero-event interpretation followed established principles [23]. Analyses were estimation-focused rather than hypothesis-testing-focused.

### 2.7. Ethical Approval

The study is conducted in accordance with the Declaration of Helsinki. Ethical approval is granted by Acıbadem University and Acıbadem Healthcare Institutions Medical Research Ethics Committee (ATADEK), 2026. Written informed consent is obtained according to local regulation and institutional policy.

## 3. Results

### 3.1. Baseline Characteristics

The cohort includes 221 ROSI evaluated cycles. The mean female age is 30.8 years, with a standard deviation of 6.4. The median age is 29 years with an interquartile range of 26 to 35. The median number of mature oocytes is 7, with an interquartile range of 2 to 10 (Table 1). Azoospermia type is distributed across cryptozoospermia, non-obstructive azoospermia, and obstructive azoospermia (Table 1). Sperm source is distributed across ejaculate, TESE, and micro TESE (Table 1). Genetic status is recorded as AZF, 47,XXY, or not recorded (Table 1). Missing genetic recordings are treated explicitly rather than excluded. The detailed characteristics of the five feasible ROSI cycles are presented in Supplementary Table S2.

### 3.2. Chain of Outcome Metrics and Attrition Pattern

**Figure 2:**
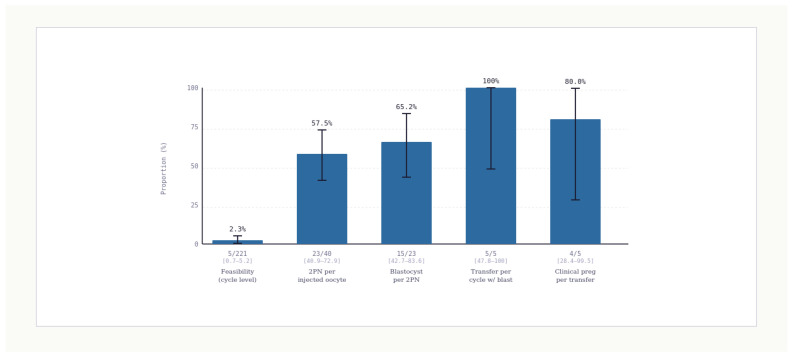
Chain of outcome metrics with exact 95% confidence intervals. Stage-specific proportions are shown for feasibility (cycle level), two pronuclei per injected oocyte, blastocyst per two pronuclei, transfer procedure per cycle with at least one blastocyst, and clinical pregnancy per transfer procedure. Exact binomial 95% confidence intervals are displayed for each stage, and denominators are labeled on the figure to emphasize conditional interpretation. Transfer and post-transfer proportions are conditional on a very small, highly selected subset (n = 5 transfers) and should not be interpreted as population-level expected success rates. Bar chart with error bars displaying stage specific proportions with exact 95% confidence intervals across the ROSI chain, with each bar labeled by its numerator and denominator.

The dominant constraint is feasibility. ROSI is feasible in 5 of 221 cycles, corresponding to 2.3%, with an exact 95% confidence interval of 0.7% to 5.2% (Table 2; Figure 2). Figure 1 summarizes the chain counts as a flow diagram and makes the feasibility bottleneck visible. This feasibility estimate answers a counseling question that is often left implicit, namely the probability that the procedure can actually be performed once it is considered.

Across feasible cycles, 40 oocytes are injected. Two-pronuclei formations are observed in 23 of the 40 injected oocytes, yielding a fertilization rate of 57.5%, with an exact 95% confidence interval from 40.9% to 73.0% (Table 2). This conditional estimate describes fertilization among those reaching injections. It does not translate directly to a per-cycle fertilization probability because the feasibility barrier occurs earlier.

Blastocyst development occurs in 15 of 23 two-pronuclei zygotes, yielding a blastocyst per two pronuclei rate of 65.2% with an exact 95% confidence interval of 42.7% to 83.6% (Table 2). This suggests that, among zygotes that fertilize, a substantial proportion progresses to blastocysts, though uncertainty remains wide because the denominator is small.

A transfer procedure occurs in all five cycles that produce at least one blastocyst corresponding to 100.0%, with an exact 95% confidence interval from 47.8% to 100.0% (Table 2). This does not imply that transfer is guaranteed in general. It indicates that transfer occurred in all observed blastocyst cycles in this cohort.

Clinical pregnancy occurred in 4 of 5 transfer procedures (80.0%), but this transfer-based rate reflects only the small, highly selected subset of cycles that successfully passed earlier feasibility, fertilization, and blastocyst development stages. For counseling purposes, the more informative overall measure is clinical pregnancy per initiated cycle, which was 4 of 221 cycles (1.8%). This shows that an apparently favorable pregnancy rate per transfer can coexist with a very low overall probability per initiated cycle when feasibility is rare.

Figure 2 presents all stage-specific proportions with exact confidence intervals and labels denominators, thereby limiting misinterpretation. This figure is intentionally structured to answer the reviewer question that often drives decision-making in experimental interventions: where exactly is the loss concentrated, and how uncertain is each estimate.

### 3.3. Pregnancy Follow-Up and Delivery Outcomes

Among 221 cases, embryo transfer was performed in 5 cases, resulting in 4 clinical pregnancies. No fetal anomaly was recorded among the clinical pregnancies (0/4). However, post-pregnancy outcome variables, including ongoing pregnancy, pregnancy loss, delivery outcome, live birth, and neonatal outcome, were not available in the retrospective study database. Therefore, pregnancy follow-up beyond the clinical pregnancy stage could not be analyzed, and the safety denominator for final obstetric and neonatal outcomes remains incomplete.

### 3.4. Subgroup Descriptive Patterns

Subgroup estimates are descriptive. They are presented to show where observed feasible cycles and pregnancies occurred in this cohort rather than to claim differences between groups.

**Figure 3:**
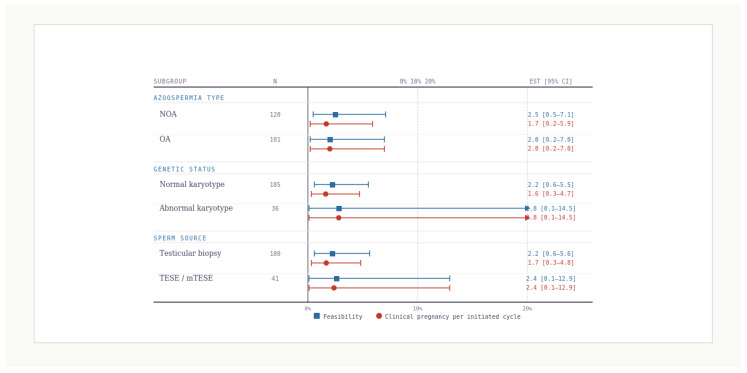
Subgroup estimates for feasibility and per cycle clinical pregnancy (descriptive). These visualized subgroup patterns should not be interpreted as evidence of subgroup effect, superiority, inferiority, or reproducible between-group difference. Descriptive subgroup estimates are presented for feasibility and clinical pregnancy per initiated cycle across azoospermia type, genetic status, and sperm source. Feasibility estimates are displayed with square markers and clinical pregnancy per-cycle estimates with circular markers, each with exact 95% confidence intervals. Results are descriptive due to sparse events and are intended to visualize uncertainty and overlap rather than support causal subgroup comparisons. Forest plot shows subgroup-specific feasibility and clinical pregnancy per initiated cycle with exact 95% confidence intervals across categories of azoospermia type, genetic status, and sperm source; squares indicate feasibility and circles indicate pregnancy per cycle.

By azoospermia type, feasibility is observed in cryptozoospermia and non-obstructive azoospermia. No feasible cycles are observed in obstructive azoospermia. These cases were included because ROSI had entered consideration within the real-world institutional pathway, not because ROSI was ultimately performed in all such cycles. Clinical pregnancies per initiated cycle occur in cryptozoospermia and non-obstructive azoospermia, while none occur in obstructive azoospermia (Table 3; Figure 3). The confidence intervals are wide, and the overlap does not support firm inference.

By azoospermia type, feasibility was observed in cryptozoospermia and non-obstructive azoospermia, whereas no feasible cycles were observed in obstructive azoospermia. These cases were included because ROSI had entered consideration within the real-world institutional pathway, not because ROSI was ultimately performed in all such cycles. Clinical pregnancies per initiated cycle were observed in cryptozoospermia and non-obstructive azoospermia, while none were observed in obstructive azoospermia. To provide greater stage-specific transparency, detailed subgroup counts across the major embryologic and clinical transitions according to azoospermia type are presented in Supplementary Table S3. The confidence intervals were wide, and the descriptive subgroup patterns should not be interpreted as evidence of between-group differences.

By genetic status category, no feasible cycles or pregnancies occurred in the 47,XXY group. One feasible cycle and one pregnancy occurred in the AZF group. Most feasible cycles and pregnancies fall into the not-recorded category, which is interpreted as an information category rather than a biological phenotype (Table 3). A sensitivity reclassification of “not recorded” genetic status as “no abnormality recorded” did not materially change the chain of outcome estimates, confirming that the primary conclusions are driven by feasibility and early fertilization rather than by the handling of missing genetic recording (Supplementary Table S1). Because the small subset of feasible ROSI cycles is clinically central to interpretation, detailed baseline and cycle-level characteristics of these five couples/cycles are now provided in Supplementary Table S2. The five feasible cycles were the only cycles in which ROSI progressed to actual oocyte injection within the available retrospective dataset.

By sperm source, feasibility is observed across ejaculate, TESE, and micro TESE categories. Clinical pregnancy per initiated cycle is observed in TESE and micro TESE. No pregnancy is recorded in the ejaculate category despite one feasible cycle (Table 3; Figure 3). Again, the purpose is transparent reporting rather than inference.

Figure 3 visualizes subgroup estimates for feasibility and per-cycle pregnancy with exact confidence intervals, emphasizing uncertainty and discouraging over-interpretation.

To improve clarity, more detailed stage-specific subgroup counts for azoospermia categories are provided in Supplementary Table S3.

### 3.5. Safety Signal and Uncertainty

No fetal anomaly was recorded among the four clinical pregnancies (0/4). This is reported as an observed absence rather than evidence of safety. With only four pregnancies, the exact binomial 95% confidence interval remains wide (0.0–60.2), meaning clinically meaningful underlying risk cannot be excluded [23,24]. The dataset also lacks long-term developmental follow-up; therefore, safety interpretation is restricted to the recorded outcomes and should be considered preliminary.

## 4. Discussion

This study provides a stage-specific map of ROSI outcomes in a high-risk real-world cohort and demonstrates that the dominant barrier is not downstream pregnancy once transfer occurs. The dominant barrier is whether ROSI can be performed at all and whether early fertilization is achieved once injection is attempted. In 221 ROSI-evaluated cycles, feasibility was observed in only five. This single fact sets the ceiling for the per-cycle probability of pregnancy and explains why overall effectiveness remains low even when pregnancy per transfer appears encouraging.

The observed attrition pattern aligns with the historical development of ROSI. Edwards and colleagues argued early that spermatid injection should be framed as an exceptional, ethically sensitive intervention with a low expected success and high uncertainty [1]. A proof of concept later established that pregnancy could occur after ROSI [2]. However, the subsequent literature reinforced instability rather than predictability. Systematic and narrative syntheses, together with newer comparative data, have continued to emphasize a marked heterogeneity, technical sensitivity, and limited reproducibility across settings [7,17,18]. The present data are consistent with that message: fertilization is not absent once injection occurs, but injection itself is rare; this combination yields sporadic successes within a narrow subset, while most evaluated cycles never enter the effective risk set for pregnancy.

A mechanistic interpretation also fits. The activation-deficit hypothesis provides a parsimonious explanation for why the chain collapses early. If round spermatids fail to trigger the physiological calcium oscillations required for oocyte activation, then the procedure’s outcome is dominated by activation barriers rather than embryo developmental competence among those reaching 2PN [8]. This explains why modern efforts often focus on activation strategies and why electrical activation has been used in contemporary series that reignited discussion [8,10,19,20,21,22]. The present study does not evaluate an activation intervention, but it supports a conclusion directly relevant to protocol optimization: if attrition concentrates at feasibility and fertilization, incremental downstream improvements will not meaningfully change the overall effectiveness unless feasibility and activation-related success improve.

The favorable downstream outcomes observed in the rare feasible cases may reflect strong biological and procedural selection rather than a reproducibly high efficacy across all ROSI candidates. Possible contributing factors include better underlying oocyte competence, more favorable female age, successful identification of the intended spermatid population, technical laboratory expertise, and the fact that cycles reaching transfer had already passed multiple upstream bottlenecks. Thus, success in these selected cases should be interpreted as proof of possibility under highly selective conditions rather than as a stable expectation for the broader candidate population.

The chain-of-outcome approach is not a cosmetic reporting choice. It directly addresses a recognized limitation in the ROSI literature: heterogeneity and inconsistent reporting weaken interpretability and complicate counseling [7]. A chain framework forces explicit denominators and avoids misleading comfort from reporting pregnancy per transfer without revealing how few cycles reached transfer. It clarifies that pregnancy per transfer is conditional on passing multiple filters and that per-cycle pregnancy reflects the integrated experience of couples entering a last-resort pathway.

The data also illustrate a common interpretive trap. Pregnancy per transfer in this cohort is 80%, but pregnancy per initiated cycle is 1.8%. This discrepancy does not imply error; it reveals selection. The transfer denominator is enriched because it includes only cycles that were feasible, fertilized, and produced blastocysts. Such enrichment can generate a high transfer-level pregnancy rate even when the overall yield is low. Similar interpretive concerns appear in earlier reports describing sporadic success among selected cases and variable performance across settings [35,36,37]. Embryo development and progression after round spermatid use may further enrich downstream denominators and inflate conditional rates relative to population-level expectations [35,36,37]. The present findings are consistent with that logic: higher downstream conditional rates likely reflect a small and selected subset that already overcame dominant bottlenecks.

Safety interpretation demands even stricter logic. No fetal anomaly was recorded among four pregnancies; this is descriptive, not conclusive. Zero-event logic makes clear that the absence of observed events in small samples cannot exclude meaningful underlying risk [23]. Exact binomial confidence intervals reinforce this by producing wide upper bounds when denominators are small [24]. The broader assisted-reproduction literature further supports caution: birth defect risk estimation depends on an adequate adjustment for confounding, and infertility and parental factors can influence observed associations [25,26,27,28,29]. More recent imprinting-focused studies likewise suggest that absolute risks are low but not necessarily null, particularly in selected ART subgroups [32,33]. These concerns are salient for ROSI because candidates are highly selected and underlying infertility is severe. Epigenetic and imprinting discussions add another reason to avoid reassurance based on small short-term series [30,31,32,33]. Contemporary cohorts reporting early childhood follow-up contribute context, yet they do not remove the need for broader surveillance because protocols, selection criteria, and laboratory practices differ across centers [34]. In the present cohort, the safety message is therefore deliberately limited: no signal is observed, but uncertainty remains wide.

From a guideline perspective, the findings support the continued positioning of ROSI as a last-resort experimental strategy. Limited feasibility in routine practice is not a minor inconvenience; it is the dominant constraint. This aligns with Practice Committee statements and newer professional guidance emphasizing experimental status, limited evidence, transparent informed consent, careful reporting, and thorough male infertility evaluation before treatment escalation [12,13,14,15,16]. The contribution of the present work is to quantify, within one real-world cohort, the stages most strongly determining overall effectiveness and to provide a reporting template that can be used across centers.

The study has limitations and strengths that must be weighed carefully. The design is retrospective and single-center, limiting generalizability and introducing potential selection and measurement biases. The number of feasible ROSI cycles is extremely small, limiting the evaluation of predictors, protocol hypotheses, or subgroup differences. However, these limitations reflect clinical reality and strengthen the case for stage-specific denominators and exact uncertainty. The study’s key strength lies in the chain framework: it separates feasibility from downstream conditional outcomes, reports exact uncertainty rather than overconfident point estimates, and provides transparent subgroup descriptions without causal overreach. Consecutive inclusion reduces selective reporting, and count-based endpoints reduce interpretive ambiguity. These findings should be interpreted cautiously. The study is retrospective, single-center, and based on a limited number of feasible ROSI cycles, which restricts precision, limits external generalizability, and precludes the robust evaluation of predictors of success. In addition, some clinically relevant variables, including detailed genetic characterization, were incompletely recorded in the retrospective database. Because only five cycles reached ROSI feasibility, each downstream percentage is highly sensitive to the success or failure of a single cycle and should therefore be interpreted as illustrative rather than stable.

Missing genetic documentation is not merely a technical missingness issue in this cohort. The “not recorded” category accounted for the most feasible cycles and most observed pregnancies, which raises the possibility that these cases differed etiologically from those with documented AZF or 47,XXY status. Accordingly, subgroup patterns by genetic category should be interpreted with particular caution, and the present sensitivity analysis cannot eliminate the residual confounding introduced by incomplete genetic characterization.

The findings have direct implications for counseling and research design. Counseling should explicitly separate the probability of being able to perform ROSI from the probability of pregnancy conditional on transfer. Programs should present chain summaries including feasibility, fertilization, and per-cycle pregnancy with uncertainty intervals. This approach gives laboratories and clinicians a clear target: if feasibility and fertilization are the main constraints, optimization should focus on cell identification, activation strategies, and protocol standardization.

Future research should move beyond isolated case series toward coordinated registries using standardized chain definitions. A multi-center registry could prespecify denominators, capture protocol-level variables relevant to activation and cell identification and follow offspring longitudinally. Even without randomized trials, standardized chain reporting would improve comparability across programs and support ethically grounded decision-making.

## 5. Conclusions

In this real-world cohort, ROSI appeared to be primarily constrained by a very low feasibility and early embryologic attrition; however, these findings require cautious interpretation given the retrospective single-center design and the very small number of feasible cycles. Downstream outcomes may appear favorable among the rare cycles reaching transfer, yet per-cycle effectiveness remained low because most cycles never entered the effective risk set for pregnancy. The absence of recorded anomalies (0/4) provides minimal reassurance because uncertainty is large and long-term follow-up was unavailable. Chain-of-outcome reporting should be adopted routinely for last-resort interventions to clarify attrition stages, improve counseling, and align protocol development with the stages most responsible for loss.

## Figures and Tables

**Table 1:** Baseline characteristics of the ROSI evaluated cohort (n = 221).

Characteristic	Value
Female age (years), mean (SD)	30.8 (6.4)
Female age (years), median (IQR)	29 (26–35)
MII oocytes, median (IQR)	7 (2–10)
Azoospermia type: cryptozoospermia, n (%)	76 (34.4)
Azoospermia type: non-obstructive azoospermia, n (%)	76 (34.4)
Azoospermia type: obstructive azoospermia, n (%)	69 (31.2)
Sperm source: ejaculate, n (%)	76 (34.4)
Sperm source: TESE, n (%)	72 (32.6)
Sperm source: micro TESE, n (%)	73 (33.0)
Genetic status: not recorded, n (%)	78 (35.3)
Genetic status: 47,XXY, n (%)	74 (33.5)
Genetic status: AZF, n (%)	69 (31.2)

Values are presented as mean (SD), median (IQR), or n (%). Percentages may not total 100% due to rounding. MII denotes metaphase II oocytes. TESE denotes testicular sperm extraction. Micro TESE denotes microsurgical TESE. “Not recorded” indicates missing genetic recording in the dataset. Baseline demographic and cycle characteristics of cycles in which ROSI was evaluated within routine clinical practice.

**Table 2:** Chain of outcome metrics for ROSI with exact 95% confidence intervals.

Stage-Specific Metric	n/N	Estimate (%)	Exact 95% CI
Feasibility (cycle level; ≥1 injected oocyte)	5/221	2.3	0.7–5.2
Two pronuclei per injected oocyte	23/40	57.5	40.9–73.0
Blastocyst per two pronuclei	15/23	65.2	42.7–83.6
Transfer procedure per cycle with ≥1 blastocyst	5/5	100.0	47.8–100.0
Clinical pregnancy per transfer procedure	4/5	80.0	28.4–99.5
Clinical pregnancy per initiated cycle	4/221	1.8	0.5–4.6
Fetal anomaly among clinical pregnancies	0/4	0.0	0.0–60.2

Exact binomial confidence intervals were computed using the Clopper–Pearson method [24]. Feasibility is defined as at least one injected oocyte within a cycle. The fetal anomaly estimate is reported descriptively; zero observed events do not imply zero risk, and uncertainty remains wide [23]. Stage-specific chain-of-outcome estimates from cycle-level feasibility through fertilization, blastocyst development, transfer, and clinical pregnancy, with exact binomial confidence intervals to quantify uncertainty.

**Table 3:** Descriptive subgroup estimates only: feasibility and per-cycle clinical pregnancy (exact 95% CI).

Subgroup Domain	Level	Feasibility (n/N)	Feasibility % (95% CI)	Clinical Pregnancy per Initiated Cycle (n/N)	Clinical Pregnancy % (95% CI)
Azoospermia type	Cryptozoospermia	2/76	2.6 (0.3–9.2)	2/76	2.6 (0.3–9.2)
Azoospermia type	Non-obstructive azoospermia	3/76	3.9 (0.8–11.1)	2/76	2.6 (0.3–9.2)
Azoospermia type	Obstructive azoospermia	0/69	0.0 (0.0–5.2)	0/69	0.0 (0.0–5.2)
Genetic status	Not recorded	4/78	5.1 (1.4–12.6)	3/78	3.8 (0.8–10.8)
Genetic status	47,XXY	0/74	0.0 (0.0–4.9)	0/74	0.0 (0.0–4.9)
Genetic status	AZF	1/69	1.4 (0.0–7.8)	1/69	1.4 (0.0–7.8)
Sperm source	Ejaculate	1/76	1.3 (0.0–7.1)	0/76	0.0 (0.0–4.8)
Sperm source	TESE	2/72	2.8 (0.3–9.7)	2/72	2.8 (0.3–9.7)
Sperm source	micro TESE	2/73	2.7 (0.3–9.5)	2/73	2.7 (0.3–9.5)

Feasibility was defined at the cycle level as the presence of at least one injected oocyte. Subgroup results are descriptive because the number of feasible ROSI cycles is small. Exact binomial confidence intervals were computed using the Clopper–Pearson method [24]. Feasibility is defined as at least one injected oocyte within a cycle. Clinical pregnancy is reported per initiated cycle within each subgroup. Descriptive subgroup estimates of cycle-level feasibility and clinical pregnancy per initiated cycle, stratified by azoospermia type, genetic status, and sperm source. “Not recorded” indicates missing genetic recording in the dataset.

## Data Availability

The data presented in this study are available on request from the corresponding author.

## Supplementary Materials

The following supporting information can be downloaded at: https://www.mdpi.com/article/10.3390/jcm15072771/s1, Table S1: Sensitivity analysis for missing genetic status coding and its impact on subgroup estimates; Table S2: Detailed baseline and cycle-level characteristics of the five feasible ROSI cycles; Table S3: Detailed stage-specific subgroup counts according to azoospermia type.
