# Supplementary material for: Round Spermatid Injection (ROSI) as a Last Resort in High-Risk Azoospermia: Chain of Outcome Metrics and Real-World Safety Signals
Source: J Clin Med. 2026 Apr 7;15(7):2771. doi: 10.3390/jcm15072771 (PMC13073379; doi:10.3390/jcm15072771)
Supplement: Supplementary file 1 [file jcm-15-02771-s001.zip › jcm-4174347-supplementary.pdf]

Journal of
Clinical Medicine

MDPI

Supplementary Table S1. Sensitivity analysis for missing genetic status coding and its impact on subgroup estimates

Scenario	Genetic status	Feasibility (n/N)	Feasibility % (exact 95% CI)	Clinical pregnancy per cycle (n/N)	Clinical pregnancy % (exact 95% CI)
Primary coding (Not recorded as separate category)	47,XXY	0/74	0.0 (0.0–4.9)	0/74	0.0 (0.0–4.9)
Primary coding (Not recorded as separate category)	AZF	1/69	1.4 (0.0–7.8)	1/69	1.4 (0.0–7.8)
Primary coding (Not recorded as separate category)	Not recorded	4/78	5.1 (1.4–12.6)	3/78	3.8 (0.8–10.8)
Sensitivity coding (Not recorded → None recorded)	47,XXY	0/74	0.0 (0.0–4.9)	0/74	0.0 (0.0–4.9)
Sensitivity coding (Not recorded → None recorded)	AZF	1/69	1.4 (0.0–7.8)	1/69	1.4 (0.0–7.8)
Sensitivity coding (Not recorded → None recorded)	None recorded	4/78	5.1 (1.4–12.6)	3/78	3.8 (0.8–10.8)

Exact binomial confidence intervals were computed using the Clopper Pearson method (Clopper and Pearson, 1934). Feasibility is defined as at least one injected oocyte within a cycle. Clinical pregnancy is reported per initiated cycle within each subgroup. This sensitivity analysis reclassifies missing genetic recording ("Not recorded") as "None recorded" to evaluate whether subgroup estimates materially depend on missingness handling.

Sensitivity analysis assessing whether subgroup feasibility and clinical pregnancy estimates change when missing genetic recording is reclassified.

Supplementary Table S2. Detailed baseline and cycle-level characteristics of the five feasible ROSI cycles.

NOA, non-obstructive azoospermia; OA, obstructive azoospermia; Crypto, cryptozoospermia; TESE, testicular sperm extraction; mTESE, microsurgical testicular sperm extraction; AZF, Y chromosome

azoospermia factor microdeletion; 47,XXY, Klinefelter syndrome karyotype; MII, metaphase II oocyte; 2PN, two-pronuclei zygote. Transfer performed was coded as yes when embryo transfer was recorded, and clinical pregnancy was coded from the source pregnancy field. Male age was not available in the dataset. The final outcome field was coded as unavailable because ongoing pregnancy, pregnancy loss, live birth, and neonatal outcome data were not available in the retrospective dataset.

Case ID	Female age	Azoospermia type	Sperm source	Genetic status	MII oocyte count	Number of injected oocytes	2PN count	Blastocyst count	Transfer performed (yes/no)	Clinical pregnancy (yes/no)	Ongoing pregnancy / live birth / loss / unavailable
Case-001	36	Crypto	TESE		4	8	6	4	yes	yes	unavailable
Case-002	38	NOA	mTESE		5	8	5	3	yes	yes	unavailable
Case-003	27	Crypto	mTESE		10	8	4	2	yes	yes	unavailable
Case-004	32	NOA	TESE	AZF	4	8	4	3	yes	yes	unavailable
Case-005	27	NOA	Ejaculate		8	8	4	3	yes	no	unavailable

Supplementary Table S3. Detailed stage-specific subgroup counts according to azoospermia type

ROSI-evaluated cycles were defined as all subgroup-specific cycles included in the retrospective cohort. Feasible cycles were operationally defined as cycles with recorded oocyte injection, because a separate structured "ROSI feasibility" variable was not available in the dataset. Blastocyst-forming cycles were defined as cycles with at least one blastocyst. Final post-pregnancy outcomes (live birth, ongoing pregnancy, or pregnancy loss) were unavailable in the dataset; therefore, the last column reports unavailable outcomes among clinical pregnancies.

Azoospermia type	ROSI-evaluated cycles	Feasible cycles	Injected oocytes	2PN embryos	Blastocyst-forming cycles / total blastocysts	Transfer procedures	Clinical pregnancies	Live birth / ongoing / loss / unavailable
Cryptozoospermia	76	2	16	10	2 / 6	2	2	0 / 0 / 0 / 2
Non-obstructive azoospermia (NOA)	76	3	24	13	3 / 9	3	2	0 / 0 / 0 / 2
Obstructive azoospermia (OA)	69	0	0	0	0 / 0	0	0	0 / 0 / 0 / 0
Total	221	5	40	23	5 / 15	5	4	0 / 0 / 0 / 4
